# Increased expression of lipocalin-type prostaglandin D_2 _synthase in osteoarthritic cartilage

**DOI:** 10.1186/ar2581

**Published:** 2008-12-18

**Authors:** Nadia Zayed, Xinfang Li, Nadir Chabane, Mohamed Benderdour, Johanne Martel-Pelletier, Jean-Pierre Pelletier, Nicolas Duval, Hassan Fahmi

**Affiliations:** 1 Osteoarthritis Research Unit, Research Centre of the University of Montreal Hospital Center (CR-CHUM), Notre-Dame Hospital, 1560 Sherbrooke Street East, J.A. DeSève Pavilion, Y-2628, and Department of Medicine, University of Montreal, Montreal, QC, H2L 4M1, Canada; 2 Research Centre, Sacré-Coeur Hospital, 5400, Gouin Boulevard West, Montreal, QC, H4J 1C5, Canada; 3 Centre de Convalescence, de Charmilles Pavillon, 1487 des Laurentides Boulevard, Montreal, QC, H7M 2Y3, Canada

## Abstract

**Introduction:**

Prostaglandin D synthase (PGDS) is responsible for the biosynthesis of PGD and J series, which have been shown to exhibit anti-inflammatory and anticatabolic effects. Two isoforms have been identified: hematopoietic- and lipocalin-type PGDS (H-PGDS and L-PGDS, respectively). The aims of this study were to investigate the expressions of H-PGDS and L-PGDS in cartilage from healthy donors and from patients with osteoarthritis (OA) and to characterize their regulation by interleukin-1-beta (IL-1β) in cultured OA chondrocytes.

**Methods:**

The expressions of H-PGDS and L-PGDS mRNA and protein in cartilage were analyzed by real-time reverse transcriptase-polymerase chain reaction (RT-PCR) and immunohistochemistry, respectively. Chondrocytes were stimulated with IL-1β, and the expression of L-PGDS was evaluated by real-time RT-PCR and Western blotting. The roles of *de novo *protein synthesis and of the signalling pathways mitogen-activated protein kinases (MAPKs), nuclear factor-kappa-B (NF-κB), and Notch were evaluated using specific pharmacological inhibitors.

**Results:**

L-PGDS and H-PGDS mRNAs were present in both healthy and OA cartilage, with higher levels of L-PGDS than H-PGDS (> 20-fold). The levels of L-PGDS mRNA and protein were increased in OA compared with healthy cartilage. Treatment of chondrocytes with IL-1β upregulated L-PGDS mRNA and protein expressions as well as PGD_2 _production in a dose- and time-dependent manner. The upregulation of L-PGDS by IL-1β was blocked by the translational inhibitor cycloheximide, indicating that this effect is indirect, requiring *de novo *protein synthesis. Specific inhibitors of the MAPK p38 (SB 203580) and c-jun N-terminal kinase (JNK) (SP600125) and of the NF-κB (SN-50) and Notch (DAPT) signalling pathways suppressed IL-1β-induced upregulation of L-PGDS expression. In contrast, an inhibitor of the extracellular signal-regulated kinase (ERK/MAPK) (PD98059) demonstrated no significant influence. We also found that PGD_2 _prevented IL-1β-induced upregulation of L-PGDS expression.

**Conclusions:**

This is the first report demonstrating increased levels of L-PGDS in OA cartilage. IL-1β may be responsible for this upregulation through activation of the JNK and p38 MAPK and NF-κB signalling pathways. These data suggest that L-PGDS might have an important role in the pathophysiology of OA.

## Introduction

Osteoarthritis (OA) is the most common joint disorder and is a leading cause of disability throughout the world [1]. It can cause pain, stiffness, swelling, and loss of function in the joints. Pathologically, OA is characterized by progressive degeneration of articular cartilage, synovial inflammation, and subchondral bone remodeling. These processes are thought to be largely mediated through excess production of proinflammatory and catabolic mediators. Among these mediators, interleukin-1-beta (IL-1β) has been demonstrated to be predominantly involved in the initiation and progression of the disease [2-4]. One mechanism through which IL-1β exerts its effects is by inducing connective tissue cells, including chondrocytes, to produce matrix metalloproteinases (MMPs), aggrecanases, reactive oxygen species, and prostaglandins (PGs) [2].

The biosynthesis of PGs involves multiple enzymatically regulated reactions. The process is initiated through the release of arachidonic acid (AA) from the cell membrane by phospholipases. Subsequently, AA is converted to an intermediate substrate PGH_2 _by the actions of cyclooxygenase (COX). Two distinct isoforms have been identified: COX-1 is constitutively expressed, whereas COX-2 is induced by various stimuli such as proinflammatory cytokines and growth factors [5]. Once formed by COX-1 or COX-2, the unstable PGH_2 _intermediate is metabolized by specific PG synthase enzymes to generate the classical bioactive PGs, including PGE_2_, PGD_2_, PGF_2_α, PGI_2_, and thromboxane [6].

There is a growing body of evidence suggesting that PGD_2 _may have protective effects in OA and possibly other chronic articular diseases. For instance, treatment with PGD_2 _enhances the expression of the cartilage-specific matrix components collagen type II and aggrecan [7] and prevents chondrocyte apoptosis [8]. In addition, we have recently shown that PGD_2 _inhibits the induction of MMP-1 and MMP-13, which play an important role in cartilage damage [9]. Thus, PGD_2 _can mediate its chondroprotective effects not only through chondrogenesis enhancement, but also through inhibition of catabolic events. PGD_2 _was also shown to exhibit anti-inflammatory properties. Indeed, increased levels of PGD_2 _are observed during the resolution phase of inflammation and the inflammation is exacerbated by COX inhibitors [10,11]. The anti-inflammatory role of PGD_2 _is supported by studies using PGD_2 _synthase-deficient and transgenic mice. The knockout animals show impaired resolution of inflammation, and transgenic animals have little detectable inflammation [12]. In addition, retroviral delivery of PGD_2 _synthase suppresses inflammatory responses in a murine air-pouch model of monosodium urate monohydrate crystal-induced inflammation [13]. Some effects of PGD_2 _can be mediated by its dehydration end product, 15d-PGJ_2 _(15-deoxy-delta12,14-PGJ_2_), which has been shown to exhibit potent anti-inflammatory and anticatabolic properties [14]. PGD_2 _exerts its effects principally by binding and activating two plasma membrane receptors, the D prostanoid receptor (DP) 1 [15] and chemoattractant-receptor-like molecule expressed on Th2 cells (CRTH2), also known as DP2 [16]. The effects of the PGD_2 _metabolite 15d-PGJ_2 _are mediated through mechanisms independent of and dependent on nuclear peroxisome proliferator-activated receptor-gamma (PPARγ) [14,17,18].

The biosynthesis of PGD_2 _from its precursor PGH_2 _is catalyzed by two PGD synthases (PGDSs): one is gluthatione-independent, the lipocaline-type PGDS (L-PGDS), and the other is glutathione-requiring, the hematopoietic PGDS (H-PGDS) [19]. L-PGDS (also called β-trace) is expressed abundantly in the central nervous system [20,21], the heart [22], the retina [23], and the genital organs [24]. H-PGDS is expressed mainly in mast cells [25], megakaryocytes [26], and T-helper 2 lymphocytes [27]. So far, little is known about the expression and regulation of L-PGDS and H-PGDS in cartilage. To better understand the role of PGD_2 _in the joint, we investigated the expressions of H-PGDS and L-PGDS in healthy and OA cartilage. Moreover, we explored the effect of IL-1β, a key cytokine in the pathogenesis of OA, on L-PGDS expression in cultured chondrocytes.

## Materials and methods

### Reagents

Recombinant human IL-1β was obtained from Genzyme (Cambridge, MA, USA). Cycloheximide (CHX) was purchased from Sigma-Aldrich Canada (Oakville, ON, Canada). SB203580, SP600125, PD98059, SN-50, and *N*-[*N*-(3,5-diflurophenylacetate)-L-alanyl]-(S)-phenylglycine *t*-butyl ester (DAPT) were from Calbiochem (now part of EMD Biosciences, Inc., San Diego, CA, USA). PGD_2 _was from Cayman Chemical Company (Ann Arbor, MI, USA). Dulbecco's modified Eagle's medium (DMEM), penicillin and streptomycin, foetal calf serum (FCS), and TRIzol^® ^reagent were from Invitrogen (Burlington, ON, Canada). All other chemicals were purchased from either Bio-Rad Laboratories (Mississauga, ON, Canada) or Sigma-Aldrich Canada.

### Specimen selection and chondrocyte culture

Healthy cartilage and synovial fluids were obtained at necropsy, within 12 hours of death, from donors with no history of arthritic diseases (n = 13, mean ± standard deviation [SD] age of 64 ± 17 years). To ensure that only healthy tissue was used, cartilage specimens were thoroughly examined both macroscopically and microscopically. OA cartilage and synovial fluids were obtained from patients undergoing total knee replacement (n = 32, mean ± SD age of 67 ± 16 years). All OA patients were diagnosed on criteria developed by the American College of Rheumatology Diagnostic Subcommittee for OA [28]. At the time of surgery, the patients had symptomatic disease requiring medical treatment in the form of nonsteroidal anti-inflammatory drugs or selective COX-2 inhibitors. Patients who had received intra-articular injections of steroids were excluded. The Clinical Research Ethics Committee of Notre-Dame Hospital (Montreal, QC, Canada) approved the study protocol and the informed consent form.

Chondrocytes were released from cartilage by sequential enzymatic digestion as previously described [29]. Briefly, this consisted of 2 mg/mL pronase for 1 hour followed by 1 mg/mL collagenase for 6 hours (type IV; Sigma-Aldrich Canada) at 37°C in DMEM and antibiotics (100 U/mL penicillin and 100 μg/mL streptomycin). The digested tissue was briefly centrifuged and the pellet was washed. The isolated chondrocytes were seeded at high density in tissue culture flasks and cultured in DMEM supplemented with 10% heat-inactivated FCS. At confluence, the chondrocytes were detached, seeded at high density, and allowed to grow in DMEM, supplemented as above. The culture medium was changed every second day, and 24 hours before the experiment, the cells were incubated in fresh medium containing 0.5% FCS. Only first-passaged chondrocytes were used.

### RNA extraction and reverse transcriptase-polymerase chain reaction

Total RNA from homogenized cartilage or stimulated chondrocytes was isolated using the TRIzol^® ^reagent (Invitrogen) in accordance with the manufacturer's instructions. To remove contaminating DNA, isolated RNA was treated with RNase-free DNase I (Ambion, Inc., Austin, TX, USA). The RNA was quantitated using the RiboGreen RNA quantitation kit (Molecular Probes, Inc., now part of Invitrogen Corporation, Carlsbad, CA, USA), dissolved in diethylpyrocarbonate (DEPC)-treated H_2_O, and stored at -80°C until use. One microgram of total RNA was reverse-transcribed using Moloney murine leukemia virus reverse transcriptase (RT) (Fermentas, Burlington, ON, Canada), as detailed in the manufacturer's guidelines. One fiftieth of the RT reaction was analyzed by real-time quantitative polymerase chain reaction (PCR) as described below. The following primers were used: L-PGDS [GeneBank: NM000954], sense 5'-AACCAGTGTGAGACCCGAAC-3', antisense 5'-AGGCGGTGAATTTCTCCTTT-3'; H-PGDS [GeneBank: NM014485], sense 5'-CCCCATTTTGGAAGTTGATG-3', antisense 5'-TGAGGCGCATTATACGTGAG-3; and glyceraldehyde-3-phosphate dehydrogenase (GAPDH) [GeneBank: NM002046], sense 5'-CAGAACATCATCCCTGCCTCT-3', antisense 5'-GCTTGACAAAGTGGTCGTTGAG-3'.

Quantitative PCR analysis was performed in a total volume of 50 μL containing template DNA, 200 nM of sense and antisense primers, 25 μL of SYBR^® ^Green master mix (Qiagen, Mississauga, ON, Canada), and uracil-*N*-glycosylase (UNG) (0.5 units; Epicentre Biotechnologies, Madison, WI, USA). After incubation at 50°C for 2 minutes (UNG reaction) and at 95°C for 10 minutes (UNG inactivation and activation of the AmpliTaq Gold enzyme; Qiagen), the mixtures were subjected to 40 amplification cycles (15 seconds at 95°C for denaturation and 1 minute for annealing and extension at 60°C). Incorporation of SYBR^® ^Green dye into PCR products was monitored in real time using a GeneAmp 5700 Sequence detection system (Applied Biosystems, Foster City, CA, USA), allowing the determination of the threshold cycle (C_T_) at which exponential amplification of PCR products begins. After PCR, dissociation curves were generated with one peak, indicating the specificity of the amplification. A C_T _value was obtained from each amplification curve using the software provided by the manufacturer (Applied Biosystems).

Relative amounts of mRNA in healthy and OA cartilage were determined using the standard curve method. Serial dilutions of internal standards (plasmids containing cDNA of target genes) were included in each PCR run, and standard curves for the target gene and for *GAPDH *were generated by linear regression using log (C_T_) versus log (cDNA relative dilution). The C_T _values were then converted to number of molecules. Relative mRNA expression in cultured chondrocytes was determined using the ΔΔC_T _method, as detailed in the guidelines of the manufacturer (Applied Biosystems). A ΔC_T _value was first calculated by subtracting the C_T _value for the housekeeping gene *GAPDH *from the C_T _value for each sample. A ΔΔC_T _value was then calculated by subtracting the ΔC_T _value of the control (unstimulated cells) from the ΔC_T _value of each treatment. Fold changes compared with the control were then determined by raising 2 to the -ΔΔC_T _power. Each PCR generated only the expected specific amplicon as shown by the melting-temperature profiles of the final product and by gel electrophoresis of test PCRs. Each PCR was performed in triplicate on two separate occasions for each independent experiment.

### Immunohistochemistry

Cartilage specimens were processed for immunohistochemistry as previously described [29]. The specimens were fixed in 4% paraformaldehyde and embedded in paraffin. Sections (5 μm) of paraffin-embedded specimens were deparaffinized in toluene and were dehydrated in a graded series of ethanol. The specimens were then preincubated with chondroitinase ABC (0.25 U/mL in phosphate-buffered saline [PBS] pH 8.0) for 60 minutes at 37°C, followed by a 30-minute incubation with Triton X-100 (0.3%) at room temperature. Slides were then washed in PBS followed by 2% hydrogen peroxide/methanol for 15 minutes. They were further incubated for 60 minutes with 2% healthy serum (Vector Laboratories, Burlingame, CA, USA) and overlaid with primary antibody for 18 hours at 4°C in a humidified chamber. The antibody was a rabbit polyclonal anti-human L-PGDS (United States Biological Inc., Swampscott, MA, USA), used at 10 μg/mL. Each slide was washed three times in PBS pH 7.4 and stained using the avidin-biotin complex method (Vectastain ABC kit; Vector Laboratories). The colour was developed with 3,3'-diaminobenzidine (DAB) (Vector Laboratories) containing hydrogen peroxide. The slides were counterstained with eosin. The specificity of staining was evaluated by using antibody that had been preadsorbed (1 hour at 37°C) with a 20-fold molar excess of recombinant human L-PGDS (Cayman Chemical Company) and by substituting the primary antibody with nonimmune rabbit IgG (Chemicon International, Temecula, CA, USA), used at the same concentration as the primary antibody. The evaluation of positive-staining chondrocytes was performed using our previously published method [29]. For each specimen, six microscopic fields were examined under × 40 magnification. The total number of chondrocytes and the number of chondrocytes staining positive were evaluated, and the results were expressed as the percentage of chondrocytes staining positive (cell score).

### Western blot analysis

Chondrocytes were lysed in ice-cold lysis buffer (50 mM Tris-HCl, pH 7.4, 150 mM NaCl, 2 mM EDTA [ethylenediaminetetraacetic acid], 1 mM PMSF [phenylmethylsulphonyl fluoride], 10 μg/mL each of aprotinin, leupeptin, and pepstatin, 1% NP-40, 1 mM Na_3_VO_4_, and 1 mM NaF). Lysates were sonicated on ice and centrifuged at 12,000 revolutions per minute for 15 minutes. The protein concentration of the supernatant was determined using the bicinchoninic acid method (Pierce, Rockford, IL, USA). Twenty micrograms of total cell lysate was subjected to SDS-PAGE and electrotransferred to a nitrocellulose membrane (Bio-Rad Laboratories). After blocking in 20 mM Tris-HCl pH 7.5 containing 150 mM NaCl, 0.1% Tween 20, and 5% (wt/vol) nonfat dry milk, blots were incubated overnight at 4°C with the primary antibody and washed with a Tris buffer (Tris-buffered saline pH 7.5 with 0.1% Tween 20). The blots were then incubated with horseradish peroxidase-conjugated secondary antibody (Pierce), washed again, incubated with SuperSignal Ultra Chemiluminescent reagent (Pierce), and, finally, exposed to Kodak X-Omat film (Eastman Kodak Company, Rochester, NY, USA). Bands on the films were scanned using the imaging system Chemilmager 4000 (Alpha Innotech Corporation, San Leandro, CA, USA), and the intensity of the L-PGDS bands was normalized by dividing them by the intensity of the β-actin band of the corresponding sample.

### 11β-PGF_2_α and PGD_2 _assays

The levels of 11β-PGF2α in hyaluronidase-treated synovial fluids and of PGD_2 _in chondrocyte supernatants were determined using competitive enzyme immunoassays from Cayman Chemical Company. Assays were performed according to the manufacturer's recommendation.

## Statistical analysis

Data are expressed as the mean ± standard error of the mean (SEM). Statistical significance was assessed by the two-tailed Student *t *test. *P *values of less than 0.05 were considered significant.

## Results

### Expressions of L-PGDS and H-PGDS in healthy and osteoarthritis cartilage

**Figure 1:**
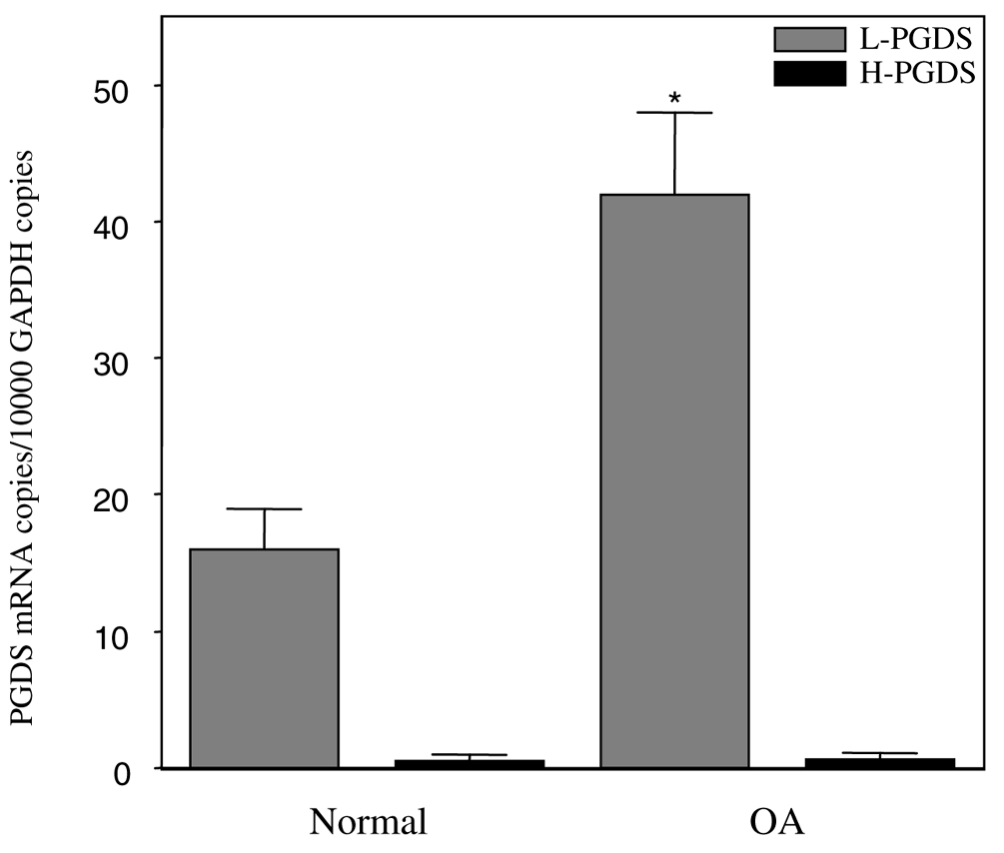
**Lipocalin-type prostaglandin D synthase (L-PGDS) and hematopoietic-type PGDS (H-PGDS) mRNA levels in healthy and osteoarthritis (OA) human cartilage**. RNA was extracted from healthy (n = 9) and OA (n = 9) cartilage, reverse-transcribed into cDNA, and processed for real-time polymerase chain reaction. The threshold cycle values were converted to the number of molecules, as described in Materials and methods. Data are expressed as copies of the gene's mRNA detected per 10,000 GAPDH copies. *P < 0.05 versus healthy samples. GAPDH, glyceraldehyde-3-phosphate dehydrogenase.

We first analyzed the levels of L-PGDS and H-PGDS mRNAs in healthy and OA cartilage using real-time quantitative RT-PCR. As shown in Figure 1, cartilage predominantly expresses L-PGDS mRNA, and its levels of expression were approximately threefold higher in OA cartilage compared with healthy cartilage. In contrast to L-PGDS, there was no statistically significant difference in the levels of H-PGDS mRNA between OA and healthy cartilage (Figure 1). In preliminary experiments, we showed that the amplification efficiencies of tested genes and GAPDH were similar. The efficiencies for the amplification of each gene and the reference were approximately equal, ranging between 1.95 and 2.

**Figure 2:**
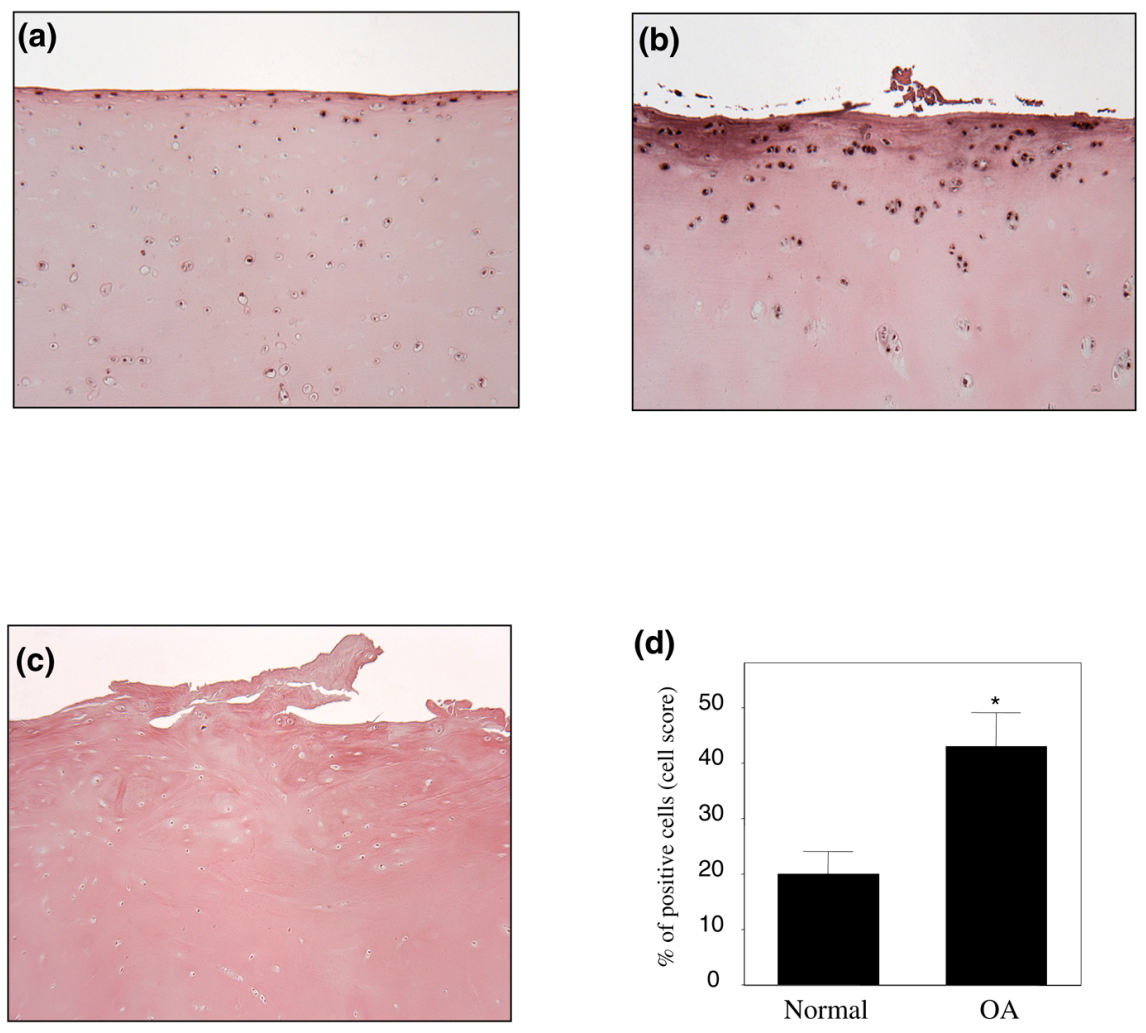
**Expression of lipocalin-type prostaglandin D synthase (L-PGDS) protein in healthy and osteoarthritis (OA) cartilage**. Representative immunostaining of human healthy **(a) **and OA **(b) **cartilage for L-PGDS protein. **(c) **OA specimens treated with anti-L-PGDS antibody that was preadsorbed with a 20-fold molar excess of recombinant human L-PGDS (control for staining specificity). **(d) **Percentage of chondrocytes expressing L-PGDS in healthy and OA cartilage. Results are expressed as the mean ± standard error of the mean of nine healthy and nine OA specimens. *P < 0.05 versus healthy cartilage.

Next, we used immunohistohemistry to analyze the localization and the expression level of L-PGDS and H-PGDS proteins in healthy and OA cartilage. As shown in Figures 2a and 2b, the immunostaining for L-PGDS was located in the superficial and upper intermediate layers of cartilage. Statistical evaluation for the cell score revealed a clear and significant increase in the number of chondrocytes staining positive for L-PGDS in OA cartilage (43% ± 6%, mean ± SEM) compared with healthy cartilage (20% ± 4%, mean ± SEM). The specificity of the staining was confirmed using antibody that had been preadsorbed (1 hour at 37°C) with a 20-fold molar excess of the recombinant protein (Figure 2c) or nonimmune control IgG (data not shown). Using several commercially available antibodies directed against human H-PGDS, we were unable to detect H-PGDS protein expression in OA or healthy cartilage. Together, these data indicate that the expression level of L-PGDS is increased in OA cartilage.

**Figure 3:**
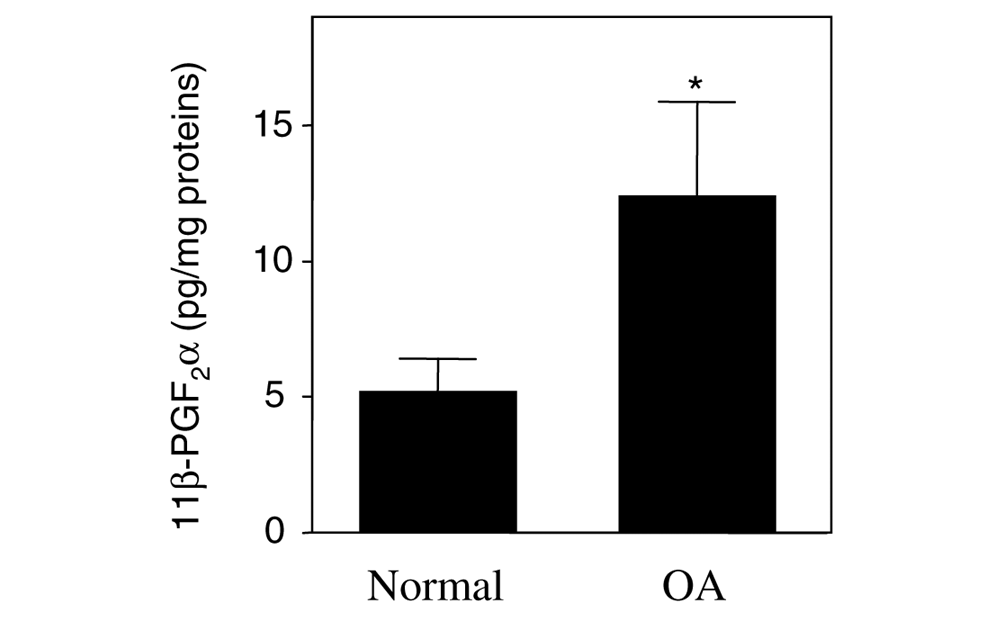
**Synovial levels of the prostaglandin D_2 _(PGD_2_) metabolite 11β-PGF_2_α**. 11β-PGF_2_α levels were measured in synovial fluids from healthy subjects and patients with osteoarthritis (OA). The results are expressed as picograms per milligram of proteins and are the mean ± standard error of the mean of 7 healthy subjects and 11 OA patients. *P < 0.05 versus healthy subjects.

To assess the level of PGD_2 _in synovial fluids from OA and healthy donors, we quantified its major stable metabolite, 11β-PGF_2_α. We measured this metabolite because PGD_2 _is unstable *in vivo *[30] and quantification of PGD_2 _in synovial fluid can be unreliable. We found a higher level of 11β-PGF_2_α in OA synovial fluid when compared with healthy synovial fluid (Figure 3), indicating that the production of PGD_2 _is higher in OA synovial fluids. Together, these data indicate increased expression and activity of L-PGDS in OA tissues.

### Interleukin-1-beta induces L-PGDS expression in chondrocytes

**Figure 4:**
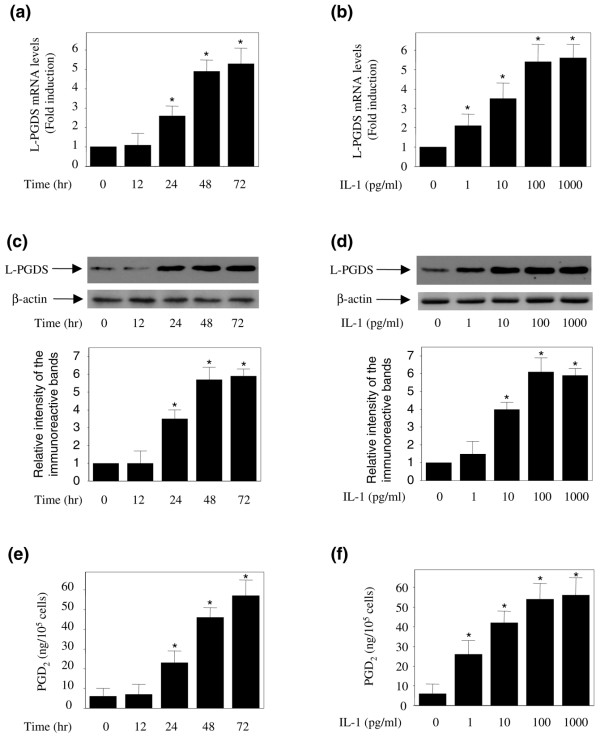
**Effect of interleukin-1-beta (IL-1β) on lipocalin-type prostaglandin D synthase (L-PGDS) expression in osteoarthritis chondrocytes**. Chondrocytes were treated with 100 pg/mL IL-1β for the indicated time periods or with increasing concentrations of IL-1β for 48 hours. **(a, b) **Total RNA was isolated and reverse-transcribed into cDNA, and L-PGDS and GAPDH mRNAs were quantified using real-time polymerase chain reaction. All experiments were performed in triplicate, and negative controls without template RNA were included in each experiment. Results are expressed as fold changes, considering 1 as the value of untreated cells, and represent the mean ± standard error of the mean (SEM) of four independent experiments. *P < 0.05 compared with unstimulated cells. **(c, d) **Cell lysates were prepared and analyzed for L-PGDS and β-actin proteins by Western blotting. Representative Western blots are shown in the upper panels. In the lower panels, the bands were scanned, and the L-PGDS band intensity values were normalized to the corresponding β-actin band intensity value. Data are expressed as fold induction, considering 1 as the value of unstimulated cells, and represent the mean ± SEM of four independent experiments. *P < 0.05 compared with unstimulated cells. **(e, f) **Conditioned media was collected and analyzed for prostaglandin D_2 _(PGD_2_) content. Results are expressed as the mean ± SEM of four independent experiments. *P < 0.05 compared with unstimulated cells. GAPDH, glyceraldehyde-3-phosphate dehydrogenase.

IL-1β plays a major role in the cartilage physiology and in the pathogenesis of OA [2]; therefore, we examined its effects on the expression of L-PGDS in cultured OA chondrocytes. Cells were treated with IL-1β (100 pg/mL) for different time periods, and the levels of L-PGDS mRNA were quantified using real-time RT-PCR. IL-1β-induced changes in gene expression were evaluated as fold over control (untreated cells) after normalization to the internal control gene, *GAPDH*. As shown in Figure 4a, treatment with IL-1β (100 pg/mL) enhanced L-PGDS mRNA expression in a time-dependent manner. L-PGDS mRNA expression started to gradually increase 24 hours post-stimulation with IL-1β and remained elevated until 72 hours. The induction of L-PGDS mRNA by IL-1β was also dose-dependent. A significant increase at concentrations as low as 10 pg/mL was observed and the maximal effect was reached at 100 pg/mL (Figure 4b). To determine whether changes in mRNA levels were paralleled by changes in L-PGDS protein levels, we performed Western blot analysis. Consistent with its effects on L-PGDS mRNA, treatment with IL-1β led to a dose- and time-dependent increase in the L-PGDS protein expression (Figure 4c, d). To establish whether the IL-1β-induced increase in L-PGDS expression corresponded with an increase in PGDS activity, we measured PGD_2 _levels in conditioned media. As shown in Figures 4e and 4f, the increased expression of L-PGDS protein was accompanied by a time- and dose-dependent increase in PGD_2 _production.

### The upregulation of L-PGDS mRNA expression in chondrocytes requires *de novo *protein synthesis

**Figure 5:**
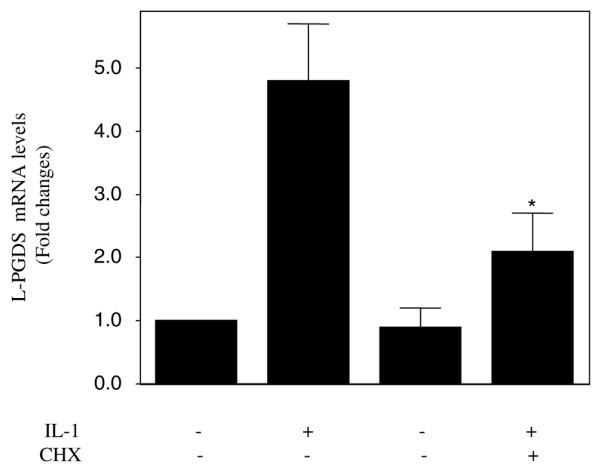
**The interleukin-1-beta (IL-1β)-induced upregulation of lipocalin-type prostaglandin D synthase (L-PGDS) mRNA expression requires *de novo *protein synthesis**. Chondrocytes were incubated with cycloheximide (CHX) (10 μg/mL) for 30 minutes prior to stimulation with 100 pg/mL IL-1β for 48 hours. Total RNA was isolated and reverse-transcribed into cDNA, and L-PGDS mRNA was quantified using real-time polymerase chain reaction. Results are expressed as fold changes, considering 1 as the value of untreated cells, and represent the mean ± standard error of the mean of four independent experiments. *P < 0.05 compared with cells treated with IL-1β alone.

The lag period required for IL-1β to induce L-PGDS mRNA in chondrocytes contrasts with those required for other IL-1β-inducible genes, the expression of which starts as early as 2 to 6 hours and reaches a maximum at 8 to 18 hours. This suggests that *de novo *protein synthesis is required for IL-1β-induced L-PGDS expression. To evaluate this possibility, we examined the impact of the protein synthesis inhibitor CHX. Chondrocytes were stimulated with IL-1β in the absence or presence of CHX, and the levels of L-PGDS mRNA were analyzed by real-time PCR. As shown in Figure 5, treatment with CHX prevented IL-1β-mediated upregulation of L-PGDS mRNA expression. This suggests that, to upregulate L-PGDS expression in chondrocytes, IL-1β must induce the synthesis of one or more proteins.

### JNK and p38 MAPKs and NF-κB pathways contribute to interleukin-1-beta-induced upregulation of L-PGDS

**Figure 6:**
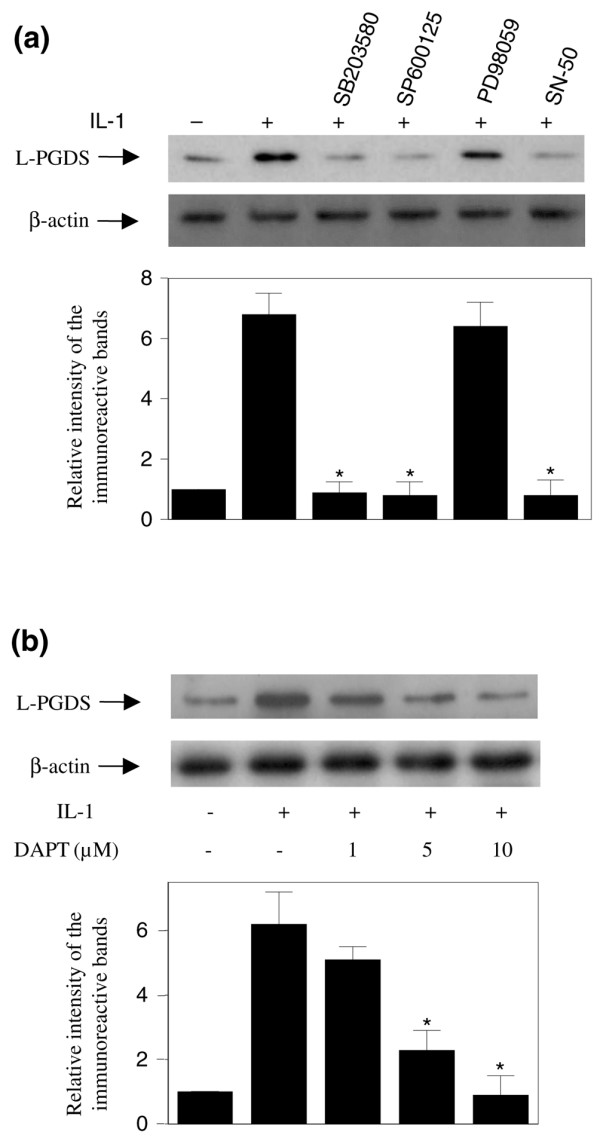
**Effect of mitogen-activated protein kinase, nuclear factor-kappa-B, and Notch inhibitors on interleukin-1-beta (IL-1β)-induced upregulation of lipocalin-type prostaglandin D synthase (L-PGDS) expression**. Osteoarthritis chondrocytes were pretreated with SB203580 (1 μM), SP600125 (10 μM), PD98059 (10 μM), or SN-50 (1 μM) for 30 minutes **(a) **or with increasing concentrations (1, 5, and 10 mM) of DAPT for 48 hours **(b) **prior to stimulation with IL-1β (100 pg/mL). After 48 hours, cell lysates were prepared and analyzed for L-PGDS and β-actin protein expression by Western blotting. Representative Western blots are shown in the upper panels. In the lower panels, the bands were scanned, and the L-PGDS band intensity values were normalized to the corresponding β-actin band intensity value. Data are expressed as fold induction, considering 1 as the value of unstimulated cells, and represent the mean ± standard error of the mean of four independent experiments. *P < 0.05 compared with cells treated with IL-1β alone. DAPT, *N*-[*N*-(3,5-diflurophenylacetate)-L-alanyl]-(S)-phenylglycine *t*-butyl ester.

IL-1β exerts its effects acting through activation of the mitogen-activated protein kinase (MAPK) (extracellular signal-regulated kinase [ERK], c-jun N-terminal kinase [JNK], and p38) and nuclear factor-kappa-B (NF-κB) signalling cascades [31-35]. To evaluate the potential contribution of these pathways in IL-1β-induced L-PGDS expression, we used specific pharmacological inhibitors. Chondrocytes were pretreated for 30 minutes with selective inhibitors for the above pathways and then stimulated or not with IL-1β for 48 hours. As shown in Figure 6a, pretreatment with the p38 MAPK inhibitor SB203580 (1 μM), the JNK MAPK inhibitor SP600125 (10 μM), or the NF-κB inhibitor SN-50 (1 μM) suppressed IL-1β-induced upregulation of L-PGDS expression. In contrast, pretreatment with the p42/44 MAPK inhibitor PD98059 (10 μM) had no effect on IL-1β-induced upregulation of L-PGDS. The concentration of the MAPK and NF-κB inhibitors used for these experiments had no significant effect on cell viability as indicated by the results of the MTT (3- [4,5-dimethylthiazol-2-yl]-2,5-diphenyltetrazolium bromide) assay (data not shown). These results suggest that the activation of JNK and p38 MAPK as well as NF-κB is essential to the induction of L-PGDS by IL-1β in chondrocytes.

The Notch signalling pathway regulates diverse cellular processes, including proliferation, differentiation, and apoptosis [36], and was reported to contribute to the regulation of L-PGDS expression [37]. To determine whether this pathway participates in IL-1β-induced L-PGDS expression in human chondrocytes, we assessed the effect of DAPT. DAPT is a γ-secretase inhibitor, which blocks cleavage of the intracellular domain of all Notch proteins, and is widely used to evaluate the effect of Notch inhibition [36]. As shown in Figure 6b, pretreatment with DAPT dose-dependently prevented IL-1β-induced L-PGDS protein expression, indicating the involvement of Notch signalling in this process. Notch inhibition was confirmed by transcriptional inhibition of its direct target gene, *Hes1 *(data not shown).

### PGD_2 _downregulated L-PGDS expression

**Figure 7:**
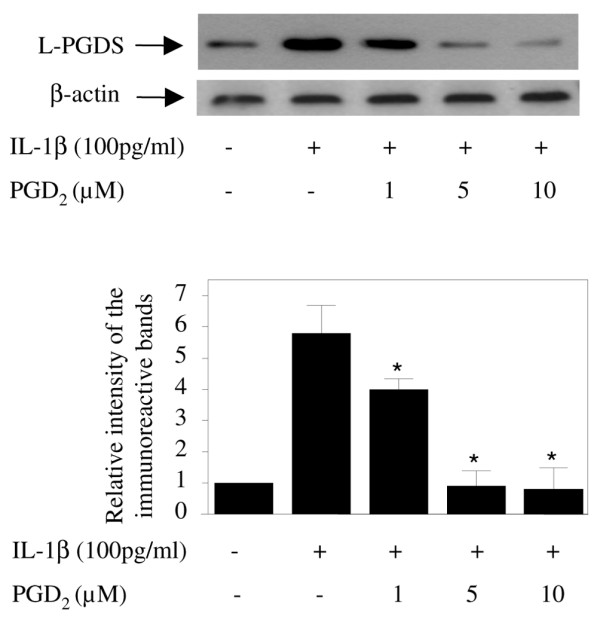
**Effect of prostaglandin D_2 _(PGD_2_) on interleukin-1-beta (IL-1β)-induced upregulation of lipocalin-type prostaglandin D synthase (L-PGDS) expression**. Osteoarthritis chondrocytes were pretreated with increasing concentrations of PGD_2 _for 30 minutes prior to stimulation with IL-1β (100 pg/mL). After 48 hours, cell lysates were prepared and analyzed for L-PGDS and β-actin protein expression by Western blotting. A representative Western blot is shown in the upper panel. In the lower panel, the bands were scanned, and the L-PGDS band intensity values were normalized to the corresponding β-actin band intensity value. Data are expressed as fold induction, considering 1 as the value of unstimulated cells, and represent the mean ± standard error of the mean of four independent experiments. *P < 0.05 compared with cells stimulated with IL-1β alone.

To further characterize the regulation of L-PGDS expression in cartilage, we examined the effect of PGD_2_, the end product of L-PGDS. Chondrocytes were stimulated with IL-1β in the absence or presence of increasing concentrations of PGD_2 _for 48 hours, and the expression of L-PGDS was evaluated by Western blotting. As shown in Figure 7, treatment with PGD_2 _dose-dependently reduced IL-1β-induced L-PGDS expression.

## Discussion

This is the first report to demonstrate the presence of L-PGDS in human cartilage and to show that its levels are elevated in OA cartilage compared with healthy cartilage. The proinflammatory cytokine IL-1β upregulated, whereas PGD_2 _downregulated, the expression of L-PGDS in cultured chondrocytes. These findings suggest that L-PGDS may be implicated in the pathogenesis of OA.

In healthy cartilage, L-PGDS immunostaining was located in only a few cells in the superficial and middle zones. By contrast, in OA cartilage, the cell score was significantly higher, particularly in cartilage areas showing significant damage (fibrillation). Given the anti-inflammatory and anticatabolic roles of PGD_2_, it is reasonable to speculate that the upregulation of L-PGDS may act as a sort of chondroprotective mechanism. Increased expression of L-PGDS was described in other diseases such as atherosclerosis [22], multiple sclerosis [38], diabetes [39] essential hypertension [40], and Tay-Sachs and Sandhoff diseases [41]. Thus, L-PGDS expression is upregulated in many pathologies.

The enhanced expression of L-PGDS in the superficial and middle zones of cartilage could potentially be due to the increased level of the proinflammatory cytokine IL-1β in these zones. Indeed, IL-1β, which plays pivotal roles in the initiation and progression of OA, has been shown to accumulate in these zones [42-46]. To prove this hypothesis, we performed cell culture experiments. Our results revealed that exposure to IL-1β led to a time- and concentration-dependent upregulation of L-PGDS expression and PGD_2 _production. The upregulation of L-PGDS expression by IL-1β was blocked by CHX, suggesting that this effect of IL-1β requires *de novo *protein synthesis and would be consistent with an indirect stimulatory mechanism.

The delayed induction of L-PGDS by IL-1β in chondrocytes is consistent with the recently reported anti-inflammatory and anticatabolic properties of PGD_2_. Indeed, the production of PGD_2 _is markedly elevated during the resolution of inflammation in carrageenan-induced pleurisy in rats, and exogenous PGD_2 _significantly reduces neutrophil levels in the inflammatory exudates [10,11]. Enhanced production of PGD_2 _was also described during the resolution phase of the wound-healing process [47]. Cipollone and colleagues [48] examined the expression of L-PGDS in atherosclerotic arteries and found lower expression of L-PGDS and higher expression of microsomal prostaglandin E synthase-1 (mPGES-1) in symptomatic plaques and found higher expression of L-PGDS and lower expression of mPGES-1 in asymptomatic ones. This suggests that the balance between PGD_2 _and PGE_2 _contributes to the pathology of atherosclerosis and that a shift toward PGD_2 _synthesis may have an anti-inflammatory role. This is supported by the observation that increased biosynthesis of PGD_2 _is associated with reduced production of PGE_2 _in several *in vitro *studies [49,50]. Recently, two separate studies demonstrated anti-inflammatory properties of PGD_2 _in an air-pouch model of inflammation induced by monosodium urate monohydrate crystals [13,51]. Moreover, H-PGDS knockout mice fail to resolve a delayed-type hypersensitivity reaction [12]. In addition to its anti-inflammatory effects, PGD_2 _was shown to induce the expression of collagen type II and aggrecan [7], to prevent apoptosis [8], and to inhibit the induction of MMP-1 and MMP-13 [52] in chondrocytes. Together, these data and those from the present study favour the hypothesis that the upregulation of L-PGDS expression in chondrocytes may be part of a negative feedback control of inflammatory and catabolic responses activated by IL-1β in the joint.

The production of PGD_2 _by chondrocytes is of particular interest since PGD_2 _is readily converted to 15d-PGJ_2_, a potent antiarthritic agent [14]. 15dPGJ_2 _downregulates the expression of a number of inflammatory and catabolic mediators involved in the pathogenesis of OA, including IL-1β, tumour necrosis factor-alpha, inducible nitric-oxide synthase, and MMPs [14]. Moreover, many *in vivo *studies support a protective effect of 15d-PGJ_2 _and other PPARγ ligands in experimental animal models of OA [53,54]. Thus, the increased expression of L-PGDS can lead to the production of a PPARγ ligand in the joint. In contrast to classical PGs, which induce their effects through binding to cell surface G protein-coupled receptors, 15d-PGJ_2 _induces most of its effects through the nuclear receptor PPARγ. We have previously shown that PPARγ expression is reduced in OA cartilage and that IL-1β downregulates its expression in chondrocytes [29], which may interfere with the protective effect of the PGD_2 _metabolite 15d-PGJ_2_. Therefore, the increased expression of L-PGDS observed in our study may represent a compensatory mechanism to counter the reduced expression of PPARγ in OA and to limit local inflammatory and catabolic responses. Also, it should be noted that 15d-PGJ_2 _can induce many of its effects independently of PPARγ [14,17,18]. In addition, PGD_2 _can directly exert protective effects in OA before being metabolized into 15d-PGJ_2_. Indeed, we have recently demonstrated that human chondrocytes express functional DP1 and CRTH-2 and that PGD_2 _downregulates MMP-1 and MMP-13 expressions through activation of the DP1 pathway [9].

To elucidate the mechanisms by which IL-1β upregulates L-PGDS expression, we evaluated the roles played by downstream signalling cascades using specific pharmacological inhibitors. We found that JNK and p38 MAPK inhibitors blocked IL-1β-induced L-PGDS upregulation, whereas an inhibitor of the ERK MAPK was without effect. We also found that NF-κB blockade caused a significant decrease in IL-1β-induced upregulation of L-PGDS protein expression. These findings support the hypothesis that the JNK and p38 MAPKs as well as the NF-κB pathways are involved in the upregulation of L-PGDS expression by IL-1β. Our results are concordant with previous reports that implicate activation of MAPKs (JNK and p38) and NF-κB in the upregulation of L-PGDS in leptomeningel cells [55], endothelial cells [56], and macrophages [57]. The activation of JNK and p38 MAPK and of NF-κB pathways in chondrocytes has been shown to cause activation of their downstream transcription factors, including activation protein-1 (AP-1) and NF-κB [31-35]. Interestingly, the promoter region of the human L-PGDS contains binding sites for NF-κB and AP-1 [55,56]. Therefore, one could speculate that upregulation of L-PGDS expression by IL-1β could be mediated by AP-1 and NF-κB. Our results also demonstrate that the Notch signalling pathway positively contributes to IL-1β-induced L-PGDS expression in chondrocytes because DAPT, a Notch signalling inhibitor, blocked this process. These findings contrast with previous data showing that the Notch pathway downregulates L-PGDS expression in the brain-derived TE671 cells [37]. The reasons for these discrepancies are presently unclear but are most likely due to cell-type differences or to differences in experimental conditions.

We also found that PGD_2 _inhibits IL-1β-induced L-PGDS expression. These results suggest that PGD_2 _may exert a negative feedback mechanism to downregulate L-PGDS expression and activity. Given that the levels of L-PGDS are elevated in OA cartilage and that IL-1β upregulated its expression in chondrocytes, it is possible that the IL-1β effect prevails over that of PGD_2 _*in vivo *during advanced stages of the disease. Indeed, the OA cartilage specimens used in this study were from donors with long-established OA. Further studies are clearly warranted to determine the expression profile of L-PGDS over the course of OA in animal models of the disease.

The concentrations of PGD_2 _used to suppress IL-1β-induced L-PGDS expression are likely to be much higher than those produced in synovial fluids. However, it should be noted that, like other eicosanoids, PGD_2 _functions as an autocrine and paracrine molecule and can readily reach pharmacological levels in the microenvironment of cells that produce it.

## Conclusion

Our study has demonstrated for the first time that L-PGDS is upregulated in OA cartilage. The proinflammatory cytokine IL-1β may be responsible for this upregulation via a mechanism that seems to involve the activation of the JNK and p38 MAPK and NF-κB signalling pathways. These results suggest that the increased expression of L-PGDS may play a protective role against articular inflammation and cartilage damage.

## Abbreviations

15d-PGJ_2_: 15-deoxy-delta12,14-PGJ_2_; AA: arachidonic acid; AP-1: activation protein-1; CHX: cycloheximide; COX: cyclooxygenase; CRTH2: chemoattractant-receptor-like molecule expressed on Th2 cells; C_T_: threshold cycle; DAPT: *N*-[*N*-(3,5-diflurophenylacetate)-L-alanyl]-(S)-phenylglycine *t*-butyl ester; DMEM: Dulbecco's modified Eagle's medium; DP: D prostanoid receptor; ERK: extracellular signal-regulated kinase; FCS: foetal calf serum; GAPDH: glyceraldehyde-3-phosphate dehydrogenase; H-PGDS: hematopoietic-type prostaglandin D synthase; IL-1β: interleukin-1-beta; JNK: c-jun N-terminal kinase; L-PGDS: lipocalin-type prostaglandin D synthase; MAPK: mitogen-activated protein kinase; MMP: matrix metalloproteinase; mPGES-1: microsomal prostaglandin E synthase-1; NF-κB: nuclear factor-kappa-B; OA: osteoarthritis; PBS: phosphate-buffered saline; PCR: polymerase chain reaction; PG: prostaglandin; PGDS: prostaglandin D synthase; PPARγ: peroxisome proliferator-activated receptor-gamma; RT: reverse transcriptase; RT-PCR: reverse transcriptase-polymerase chain reaction; SD: standard deviation; SEM: standard error of the mean; UNG: uracil-*N*-glycosylase.

## Competing interests

The authors declare that they have no competing interests.

## Authors' contributions

NZ conceived the study and designed and carried out cell and real-time RT-PCR experiments and some immunohistochemistry experiments. NC contributed to the study design and carried out immunoassays and some cell experiments. XL carried out some cell experiments and data analysis. MB participated in the study design and data analysis. JM-P, J-PP, and ND helped to obtain tissues and participated in the study design and some immunohistochemistry experiments. HF conceived, designed, and coordinated the study, carried out some cell experiments, and drafted the manuscript. All authors read and approved the final manuscript.
